# Supplementary material for: Predictors of underweight among adult patients receiving antiretroviral therapy in Bishoftu general hospital, central Ethiopia: Case-control study
Source: PLoS One. 2023 Sep 21;18(9):e0291602. doi: 10.1371/journal.pone.0291602 (PMC10513338; doi:10.1371/journal.pone.0291602)
Supplement: Attachment — Submitted filename: Point by point response to editor.docx [file pone.0291602.s002.docx]

October 14, 2022

To Editor,

**Subject: Response to editors’ comments**

Dear editor, it is our pleasure to respond to editors’ requests. We have completed and attached both the ‘clinical studies checklist’ and ‘STROBE checklist’ with the revised manuscript. Below is more information on the points completed on the ‘clinical studies checklist’.

Kindly regards,

Dawit Wolde Daka

Corresponding Author

E-mail: [dave86520@gmail.com](mailto:dave86520@gmail.com)

1. Did you obtain ethics approval for this study?

**Response:** Yes, we have obtained ethics approval and uploaded the copy.

2. If your study involved human participants, please report in the Methods section when participants were recruited for the study.

**Response:** As clearly described in the methods part (lines # 110 and 116-117), the study was conducted from April to May 2022; and the participants were recruited in a similar period.

3. If you are reporting a study of medical records or archived samples, please report in the Methods section the date range in which human subjects’ data/samples were collected and the date(s) when you conducted this study.

**Response:** As clearly described in the methods (Line #168), the records of patients who have been interviewed and anthropometric measurement taken was reviewed. Record reviews were done during the data collection period.

4. Please specify in the Methods section whether authors had access to information that could identify individual participants during or after data collection.

**Response:** As clearly described in the ethical consideration section (line # 220-222), authors had no access to information that identifies individual patients because personal identifiers were not recorded and unique codes were used to identify study participants.

5. If you are reporting an observational study – i.e. cohort, case-control, and cross-sectional studies – we recommend that the work is reported as per the requirements of the STROBE guidelines and that you provide a completed STROBE checklist as a Supporting Information file with your submission.

**Response:** We ensure that the research work was reported as per the requirements of STROBE guidelines and we have attached the completed STROBE checklist.

6. Please ensure that the author list and Corresponding Author entered in Editorial Manager match the author list and Corresponding Author in your manuscript file.

**Response:** We have checked that the author list on the manuscript and editorial manager were consistent.
