# Supplementary material for: Predictors of underweight among adult patients receiving antiretroviral therapy in Bishoftu general hospital, central Ethiopia: Case-control study
Source: PLoS One. 2023 Sep 21;18(9):e0291602. doi: 10.1371/journal.pone.0291602 (PMC10513338; doi:10.1371/journal.pone.0291602)
Supplement: Attachment — Submitted filename: Comments- plos one.docx [file pone.0291602.s003.docx]

**Comments**

1. In the case using abbreviation/acrimony in the first place use full word then abbreviation in Bereket.

Correct starting from abstract

2. In result section - Monthly income of patients <1000 birr (aOR=10.18, 95% CI: 3.31, 31.26) and 1000-

2000 birr (aOR=5.56, 95% CI: 2.33, 13.24)

Comment – aOR- Correct with AOR and you categorization of income without logical reasoning that is why AOR Very wide need adjustment.

3. In result section-Tables (2-5)title not incomplete example table 2. Clinical and immunological characteristics not incomplete should include study area and study period.
4. In result section line 290-295- In the multivariable logistic regression, variables including monthly income, presence of 292 caregiver, eating problems, dietary counseling, dietary diversity score, and khat chewing have 293 shown statistically significant associations with underweight. Though the remaining variables 294 have showed associations in the binary logistic regression with a p-value

Comment – binary include bivariate & multivariable regression analysis, based on this concept the above interpretation is wrong – the author should set criteria for those variables from bivariate analysis to multivariable analysis. i.e. p-value<0.05 is not serve as a criteria for bivariate?
